# Massage in Veterinary Therapeutics

**Published:** 1882-10

**Authors:** 


MASSAGE IN VETERINARY THERAPEUTICS.—The use of rubbing, kneading and similar movements has been rapidly gaining in favor of late. Especially is this the case among the German veterinarians connected with the army.

Dr. Paul Marggraff has contributed an interesting review of the matter to the Adam's *Wochenschrift für Thierheilkunde*.

For easier reference and description, the different forms of massage have received different names:

1. There is first the *effleurage*, or superficial rubbing, which consists in giving long, gentle strokes of the hand from the periphery toward the centre. This motion empties the superficial veins and lymphatics. It is very useful in bruises and sprains of the joints.

2. Next is *massage à friction*, which is a more active application.

It consists in similar but firmer strokes, from periphery to centre, combined with circular movements around the part or limb. The hand makes strong mechanical pressure.

As might be inferred, these movements are more applicable when inflammatory products have already been thrown out. They are most useful, therefore, in long-standing troubles, galls, chronic inflammation, etc.

3. *Petrissage*, or kneading, consists, as its name implies, of relaxing, catching hold of individual parts, pressing, squeezing, pinching, etc. Its action is much like that of friction massage (No. 2), but is deeper.

4. *Tapoltement*, or beating, consists in striking the diseased part with the fist or the edge of the open palm.

It is used in paralysis, not of central origin, in order to irritate the nerves and muscles.

Massage is most applicable in joint diseases. That it has a notable mechanical effect here is shown by an experiment of Mosengeil's. He took rabbits and injected India ink into various joints. Some of the joints he massaged, others not. He then opened the injected joints. Those that had been rubbed were almost free from ink, which could be seen coloring the out-going lymphatics for some distance. In the other joints the ink was found in abundance.

Pure glycerine is recommended as an agent to be used in massage, rather than sweet oil, which soon gets rancid. As a rule, massage should be done twice daily for from five to twenty, or in chronic troubles, thirty minutes each time. After the rubbing it is sometimes well to lead the animal about quietly for five or ten minutes, then cover the parts with warm and moist applications.

Massage is one of those forms of treatment which will give good or no results, according as it is carefully and conscientiously used.

